# Assessment of banana fruit handling practices and associated fungal pathogens in Jimma town market, southwest Ethiopia

**DOI:** 10.1002/fsn3.591

**Published:** 2018-02-08

**Authors:** Chala G. Kuyu, Yetenayet B. Tola

**Affiliations:** ^1^ Department of Post‐harvest Management Jimma University College of Agriculture and Veterinary Medicine Jimma Ethiopia

**Keywords:** banana fruit, handling practice, postharvest fungal pathogens

## Abstract

Banana fruits are highly perishable and affected by different microbial contaminates because ripe bananas are very perishable. One of the most important factors causing great economical loss of banana fruits is postharvest fungal diseases caused by lack of proper handling along postharvest chains. In line with this, the study was carried out to assess banana fruits handling practices and identify the major causal agents of postharvest fungal disease of banana fruits in Jimma town of bishishe market. Assessment was carried out using purposive sampling to select the study area, followed by simple random sampling to collect information on handling practices through observation and interview using pretested questioners. The survey result revealed that losses of banana fruits due to spoilage and physical injury were common problems for all fruit vendors. Practices such as lack of sanitation, temperature management, and improper packaging and transportation problems were identified among the common causes for observed losses. A total of 48 fruits were purposively selected from open market, wholesales and retailers and fruit damage, disease incidence, disease severity and identification of diseases causing fungal pathogen were conducted under laboratory. The highest fruit damage (56.2%) was recorded in sample taken from retailers’ shop, and the associated disease incidence and severity were 54.2% and 34%, respectively. Morphological identification of pure culture revealed that anthracnose caused by *Colletotrichum musae* and crown rot caused by fusarium spp. are the most important disease causing agents and result in large percentage of fruit loss in Jimma town of bishishe market.

## 1 INTRODUCTION

Banana is one of the most widely grown tropical fruits, cultivated over 130 countries, along the tropics and subtropics of Capricorn. It is the second largest produced fruit after citrus, contributing about 16% of the world's total fruit production and the fourth most important staple food crops in the world after rice, wheat, and maize (Ellyn, 2011). According to FAOSTAT (2014), the major banana‐producing countries that accounted for about 75% of total banana production are located in the developing world. In Ethiopia, it is a most important fruit in terms of both production and consumption (Woldu, Mohammed, Belew, Shumeta, & Bekele, 2015).

Banana fruits are highly nutritious and easily digestible than many other fruits (Mohapatra, Mishra, & Sutar, 2010). Its wide consumption is due to its sensory characteristics, particularly its attractive texture and flavor make banana popular by the consumers (Robinson and Sauco, 2010). Moreover, it has high caloric contribution leading to high demands mainly by developed countries which account for nearly 70% of world's consumption (FAO, 2012; Vazquezshy, Karina, Adriano‐Anaya, Salvador‐Figueroa, & Ov, 2012). It also contains low‐fat, excellent source of dietary fiber, vitamin C, vitamin B_6_, and manganese (Vazquezshy et al., 2012). The presence of potassium and fiber in large amounts in bananas may help combat atherosclerosis, which can lead to heart attack and stroke (Ellyn, 2011). Almost all types of bananas produced in Ethiopia are consumed fresh and play an important role in feeding the low‐income families as well as providing a source of income to them. The fact that it produces fruit throughout the year adds to its importance as a cash crop in the growing region (Daniel, 1999; Seifu, 1999). There is also a vast potential in internal market for bananas, primarily in densely populated urban areas and in export market as the country is located close to important markets such as Saudi Arabia, Djibouti, and Somalia (Tilahun, Osthoff, & Steyn, 2012).

Although banana fruits are highly demanded as nutritious and economically important fruits, they experience a different marketing problem (El‐Naby, 2010). One of the limiting factors that influence the fruits’ economic value is its relatively short shelf‐life caused by postharvest pathogens attack. The fruit contains high levels of sugars and nutrients element, and their low pH values make them particularly desirable to fungal decayed (Singh & Sharma, 2007). It is estimated that in average, about 20%–25% of the harvested banana fruits are decayed by different fungi during postharvest handling, and everyday 1.6 million bananas are thrown in developing countries (Idris, Ibrahim, & Forsido, 2015).

This fungal infection may occur during the growing season, harvesting, handling, transport and postharvest storage and marketing conditions, or after purchasing by the consumer (Warton, Wills, & Ku, 2000). Physical damage to the peel induced during handling and storage predisposes banana to be attacked by decay‐causing pathogens (Deka, Choudhury, Bhattacharyya, Begum, & Neog, 2006). Moreover, during storage, it can develop many postharvest diseases that affect the quality of the fruit including anthracnose and crown rot. The genus *Colletotrichum* and its teleomorph *Glomerella* are considered to be the major banana fruit pathogens worldwide. They cause significant economic damage to banana fruits in tropical, subtropical, and temperate regions (Bailey & Jeger, 1992). Therefore, gentle handling and appropriate storage conditions are needed along postharvesting chain to minimize mechanical damage and reduce subsequent wastage due to microbial attack (Warton et al., 2000).

Although the farmers around Jimma town in southwestern part of Ethiopia have great potential to produce high‐quality bananas, the postharvest handling and marketing practices are not to the standard. Losses of banana fruits both in quantity and quality occurring between harvesting and final utilization are extensive. Bunches are piled up high to maximize loads and compensate for transport cost (Mulualem, Jema, Kebede, & Amare, 2015). Insufficient packing, overloading, and bulk transport in the local market all together are expected to expose bananas to damage leading to deterioration in quality resulting in losses. However, little is understood about the impact of mishandling and associated fungal decay of the fruits. Furthermore, no extensive study has been carried out so far to identify and quantify the extent of postharvest losses due to mishandling practices and fungal decay. Hence, there is a vital need to understand the influence of handling practices during banana fruit marketing on the postharvest losses due to fungal pathogens. Therefore, this study aimed to assess various postharvest practices influencing postharvest losses and identify fungal pathogens responsible for the major postharvest decay of banana fruits in the study area.

## 2 MATERIALS AND METHODS

### 2.1 Description of the study areas

The assessment was carried out in Jimma town of Bishishe market. Bishishe is small market in Jimma town where fruits, vegetables, and cereals are sold. The laboratory evaluation was conducted at Jimma University College of Agriculture and Veterinary Medicine, Pathology laboratory.

### 2.2 Data collected

#### 2.2.1 Banana fruits handling practices in the Jimma town market

To collect data regarding handling practices, all the possible market places (open market up to wholesaler) were assessed and interviewed. Two‐stage sampling techniques were employed to select specific banana sellers and traders of the study area. In the first stage, Bishishe market was purposively selected based on the fact that it is the only place in Jimma town where banana fruits are sold in large amounts. In the second stage, simple random sampling method was followed, and a total of 50 respondents that represented five of wholesalers, 25 of retailers, and 20 of farmers selling banana fruits in open market were selected using Yamane 1967) sample size determination formula. The selected respondents were interviewed about postharvest handling, sanitation in the market places, and storage practices using self‐administrated questioners approach. Additionally, during data collection, temperature of the marketing area was recorded three times in a day (morning, afternoon, and evening) for consecutive 5 days, and the temperature of 1 day was averaged.

#### 2.2.2 Fruit damage, disease incidence, and severity assessment

To assess percentage of fruit damage and test for fungal pathogen contamination, representative samples (a total of 48 fruits) composed of both injured and apparently healthy looking were sampled purposively based on the volume sell from the farmers selling fruits in open market (*n* = 16), wholesalers (*n* =16), and retailers (*n* = 16) with three replications. All the fruits were transported using truck to the laboratory and stored at room temperature for further evaluation of fruit damage and quiescent (latent) infection of the pathogens.

The infection of fungal pathogens was identified using different approaches such as looking at the appearance of decayed fruit, including the color sign of the pathogen spore or fruiting bodies, and location of infection sites. Following the identification of infections, disease incidence was calculated as number of infected fruits showing any single symptom out of total number of banana fruits sampled (Ogbo & Oyibo, 2008).

$$\text{Percentage of disease incidence}=\frac{\text{Number of infected fruits}}{\text{Total number of fruit samples}}*100$$

Percentages of fruit damage were assessed and calculated using the following equation.

$$\text{Percentage of fruit damage}=\frac{\text{Number of damaged fruits}}{\text{Total number of fruit samples}}*100$$

The disease severity evaluation was undertaken by observing the fungal symptom record of disease levels according to the infected surface area on the fruits. It was measured on a 1–6 scale in which no infected surface area scored 1, whereas the infected surface areas of >0%–5%, >5%–25%, >25%–50%, >50%–75%, and >75% scored 2, 3, 4, 5, and 6, respectively (Duamkhanmanee, 2008). The percent severity index of fungal infection was then estimated from the numerical ratings of the total samples using the following formula.

$$\begin{array}{cc} \text{Percent}\; & \text{severity index}\\ & =\frac{\text{Sum of numerical ratings}}{\text{Total number of fruit examined*maximum grade}}*100 \end{array}$$

#### 2.2.3 Isolation and identification of fungal pathogens associated with banana fruit

The samples collected from different banana vendors were first washed in tap water and then the fruits that displayed symptoms of fungal infection were selected for fungal isolation. The tissues were cut from active lesions surface of the fruits and surface sterilized by soaking in freshly prepared NaOCl (3% w/v) for 3 min. After three serial washings in sterile distilled water, tissues were placed (four pieces per plate) on Potato Dextrose Agar (PDA) and incubated at 25°C in the incubator for 7 days. The colonies emerged from each plated fruit tissues were purified and subcultured on the PDA media after 7 days. The plates were incubated at 25°C under similar conditions, and the setups were observed until the organisms became fully grown. Single‐spore cultures of the fungus were then prepared on PDA slants in test tubes, and the identification and characterization of the fungal isolates were carried out based on cultural and morphological structures described in Marasas, Miller, Riley, and Visconti (2001).

## 3 RESULTS AND DISCUSSIONS

### 3.1 Socio‐demographic characteristics of the study area

Among 50 banana vendors interviewed, their gender, age, marital status, and educational levels were quantified (Table 1).

**Table 1:** Socio‐demographic characteristics of banana fruits vendors in Jimma town market

Socio‐demographic characteristics of banana fruit vendors		Frequency	Percentage
Sex	Male	14	28
	Female	36	72
Age	<25	19	38
	25–35	13	26
	35–45	10	20
	>45	8	16
Educational level	Literate	23	46
	Illiterate	27	54

In gender category, 14 (28%) of respondents were males and rest of them were females and indicated that the females were involved much in banana fruits sale. Among the age groups, the maximum of 19 (38%) members were <25 years of age followed by 25–35 years and 35–45 years of age, and the minimum of 8 (16%) vendors belong to >45 years of age. The level of education varies among the gender and age categories, 23 of them were illiterates, 27 of them were in various levels (elementary to high school) education. More than 75% of literate respondents have awareness on the influence of improper handling practices on the quality of banana fruits. Similar observation was made by Olayemi, Adegbola, Bamishaiye, and Awagu (2012) who stated that peoples on secondary educational levels can easily understand the postharvest handling practices more than peoples on primary educational levels. Babalola (2011) also reported that education enables to understand the effect of handling practices on the postharvest loss of the produces and leads to better handling practices than illiterate.

### 3.2 Banana fruits handling practices in Jimma town market

Actual observation and assessment results indicated a wide range of mishandling practices that favor fungal developments and results in fruit losses. Postharvest banana fruits fungal developments and associated losses could be related to the following mishandling practices.

#### 3.2.1 Means of banana fruits transportation to the market and packaging materials

**Figure 1:**
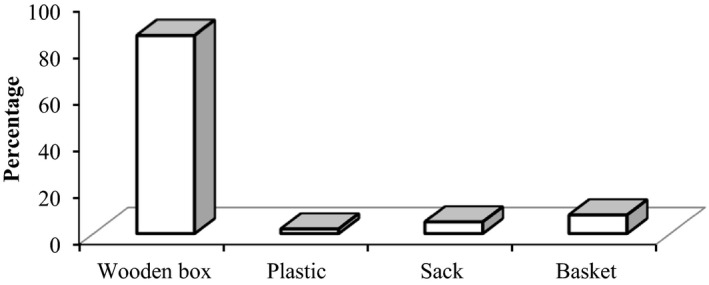
Percentage of respondents handling banana fruits using different packaging materials

Most of the banana fruits sold in Jimma town was brought from distant places using different packing materials and means of transportations (Debela, Daba, Bane, & Tolessa, 2011). About 85% of the respondents were used wooden boxes for packaging banana fruits, whereas the remaining 15% of them were used basket, plastic, and sack (Figure 1). We observed that the wooden boxes they have been used were too big and too rough to provide protection, and much of the fruits on the bottom of the wooden boxes were crushed and typically discarded before sale. Similar observation was made by Mulualem et al. (2015) who reported a high mechanical injury (50%) to bananas packed in wooden crate which could be due to compression and surface bruising damages.

**Figure 2:**
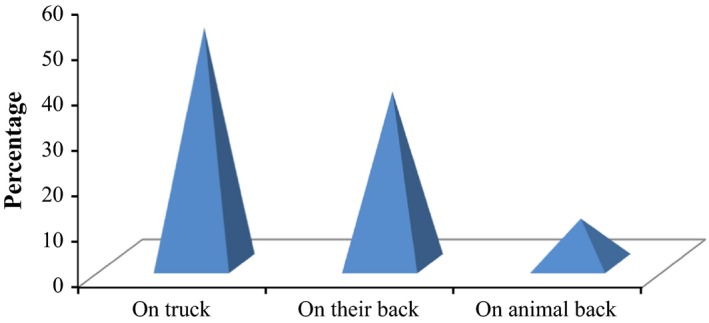
Percentage of respondents transporting banana fruits using different means of transportation

The interview result (Figure 2) indicated that 26 (52%) of the respondents transport their banana fruits to Jimma town market using truck usually for those fruits transported from longer distance. About 19 (38%) of respondents use their back and the rest of them use drought animals (donkey, horse or mule). More than 50% of the respondents criticized that, the mode of transportation is unhygienic and bananas are mixed with other fruits with less care for their damage. This could be an indication that vehicles and other commodities transported with the fruits could be a source of potential contamination. It was observed that for banana fruits transported from longer distance by truck, there was largest percentage of fruit damage because fruits transported under heavy compression force on pumper roads for long time while exposed to direct sunlight and wind. Moreover, observations were made for fruit damage between fruits packed in different packaging materials, and highest percentages of fruit damage were observed in plastics and sack in comparison with wooden boxes and basket.

#### 3.2.2 Lack of temperature management

The temperatures of the marketing area were recorded for five consecutive days and ranged from 27 to 38°C. The recorded temperature was much higher than recommended temperature for quality maintenance of the banana fruits.

**Figure 3:**
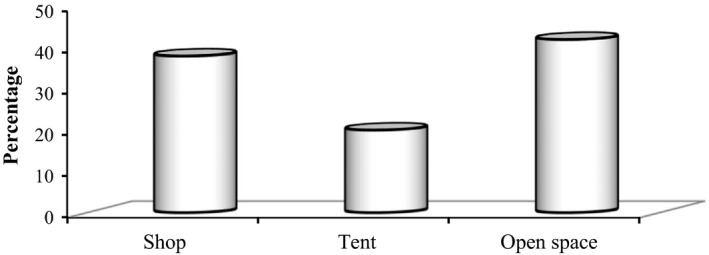
Percentage of respondents selling banana fruits in different marketing places in study area

**Figure 4:**
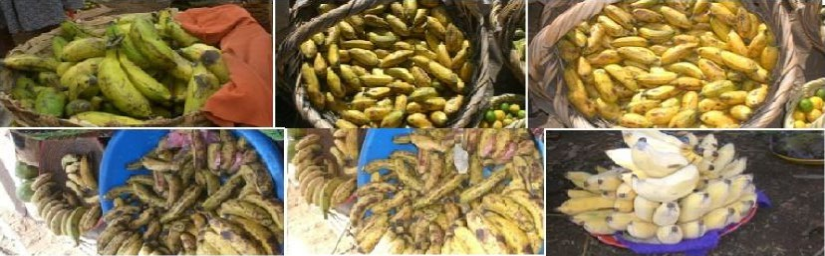
Banana fruits sold in open space without any temperature management

Most (42%) of the respondents interviewed (Figure 3) simply left the fruits exposed to ambient conditions as indicated in Figure 4 which were typically 10–20 degrees higher than the maximum handling temperatures recommended for the fruit (15°C). Twenty (20%) of the respondents sell in tent‐like shade structures, and only 38% of them were used shop intended for this purpose.

The observed temperatures of the marketing area were two to threefold higher than the optimum postharvest handling temperatures of banana fruits, and hence, shelf‐life of the fruits would be theoretically only one‐half or even one‐quarter of the potential (Gultie, Sahile, & Subramanian, 2013).

#### 3.2.3 Sanitation status of the market area

It is observed that all the banana marketing areas were not functional only for fruits but different commodities including cereals were stacked together with the fruits. In 56% of the fruit marketing shops, banana fruits were placed with other fruits together in the same container which may lead to cross‐contamination. Responses about the storage period over which the banana fruits sold safely were asked and the result indicated that 65.6% of shops stored only for 4 days in average, whereas 34.4% of banana shops stored the fruits only for 2 days in average. This indicated that the fruit vendors could not able to store the fruit even for 1 week. This might be due to spread of fungal infection as a result of lack of sanitation and presorting to remove decayed banana fruits before displaying to the market and in the shops.

All the respondents indicated that always receive physically injured and healthy fruits in the same container from the distributors. Among the banana fruit vendors, only 22 (44%) of them store damaged and spoiled fruits separately (Table 2). Therefore, there is a possibility of cross‐contamination of healthy fruits with fungal pathogens (Jennylynd, 2006). During the time of observation, 80% of the marketing areas were full of pests majorly with insect and different dirty matters. It is also observed that about 34 (68%) of the banana fruit marketing areas were contaminated by horse and donkey dung.

**Table 2:** Interview result about problems related to market and market area/shop

Interview statements	Yes	No
	Frequency (%)	Frequency (%)
Have you stored injured fruits and healthy fruits separately?	22 (44)	28 (56)
Are the area and the shop free from dust and pests?	10 (20)	40 (80)
Is the area free from mud, animal dung or wastes?	16 (32)	34 (68)
Have store banana with other commodities?	28 (56)	22 (44)
Can you store the fruit in your shop for a week?	0	50 (100)

#### 3.2.4 Percentage of fruit damage

**Figure 5:**
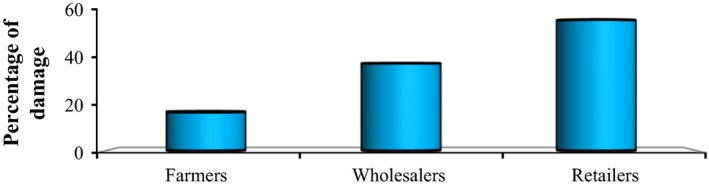
Percentage of fruit damage from different fruit handlers in Jimma town market

The percentage of fruit damage at the market varied between fruit handlers (Figure 5). The result indicated that the maximum damage (56.2%) was observed in sample taken from retailers shop, and the minimum (16.6%) was recorded from the sample taken from farmers selling their banana fruits in the market.

Different reasons were indicated as major causes for damage, and among these, negligence in good handling during harvesting and on farm, lack of proper transportation, less care during loading and unloading and etc. More than 40 (80%) of the respondents told that banana fruits sold in Jimma market are transported from long distance on bumper road and overloaded in the truck. This might induce mechanical damage on the fruits and as a result induce fungal infections. During the study, we observed that bunches are piled high on the floor during ripening process, and there is a possibility of physical damage for bunches due to overloading one on another. Moreover, there is no control mechanism for temperature and humidity in the ripening room. They simply burn gasoline as a source of heat and ethylene for 24 hrs under enclosed condition to trigger ripening process. This may cause deterioration of product trough enhancing physiological process of fruit and creating favorable environment for disease development by weakening the fruit cell wall (Eduardo, 2012).

### 3.3 Incidence and severity of fungal pathogens

The mean percent of disease incidence and severity varied between banana fruit handlers (Table 3). The maximum incidence (54.2 ± 5.2) was recorded in sample taken from retailers shop followed by sample from wholesaler (32.3 ± 3.12). In the same manner, the mean percent of disease severity was maximum at retailer's shops and minimum in sample taken from farmers (Table 3).

**Table 3:** Percentage of disease incidence and severity from different fruit handlers in Jimma town market

Fruit handlers	Incidence (%)	Severity (%)
Farmers	12.10 ± 1.40	5.6 ± 0.40
Wholesalers	32.30 ± 3.12	23.7 ± 2.30
Retailers	54.20 ± 5.20	34.0 ± 2.56
Overall mean	32.87 ± 3.24	21.1 ± 1.75

The mean percentage of disease incidence and severity results are in line with percentage of fruit damage recorded. This indicated that mechanical damage during handling at different stages along postharvest chain predisposes the fruits for fungal pathogens (Hailu, Workneh, & Belew, 2012). The difference in terms of fruit damage, percent disease incidence, and severity between fruit handlers could be due to storage. Similar result also indicated in Debela et al. (2011) who worked on identification of major causes of postharvest losses among selected fruits in Jimma zone.

### 3.4 Identification and characterization of fungal pathogens associated with banana fruit

A total of 146 fungal isolates grouped in two genera were recovered from banana fruit samples collected from three fruit handlers (farmers, wholesalers, and retailers) of Jimma town market. They were identified on the basis of their cultural and morphological structures such as shapes and sizes of macroconidia and microconidia, and colony color. The cultural and morphological investigations on the samples revealed that *Collectotrichum musae* and *Fusarium spp* were the most frequently observed, respectively (Figures 7 and 8). About 84% of colonies counted were contributed by *Collectotrichum* spp., and about 16% of colonies counted were accounted by *Fusarium* spp. In the similar manner, Zakaria, Sahak, Zakaria, and Salleh (2009) stated that *Collectotricum* are the most commonly associated with anthracnose diseases of different banana cultivars.

**Figure 6:**
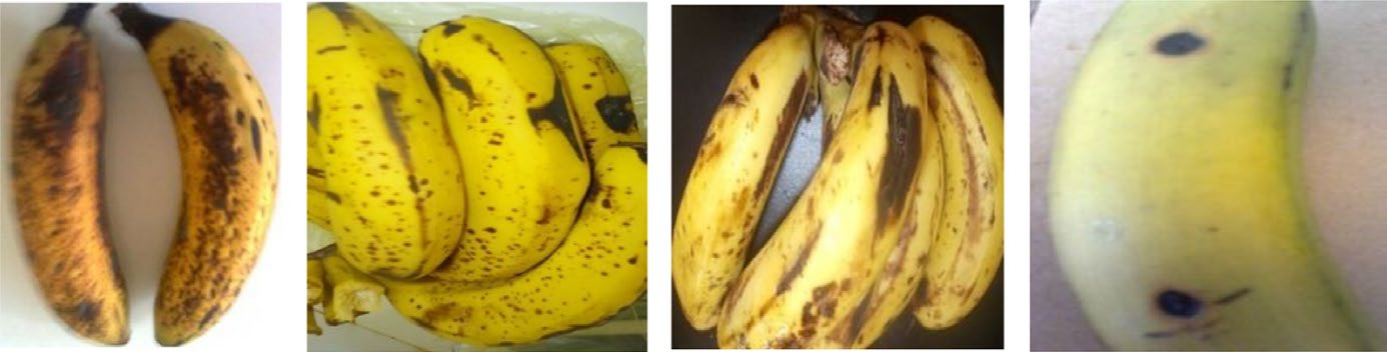
Fruit sample taken to laboratory for isolation and identification of fungal pathogens

**Figure 7:**
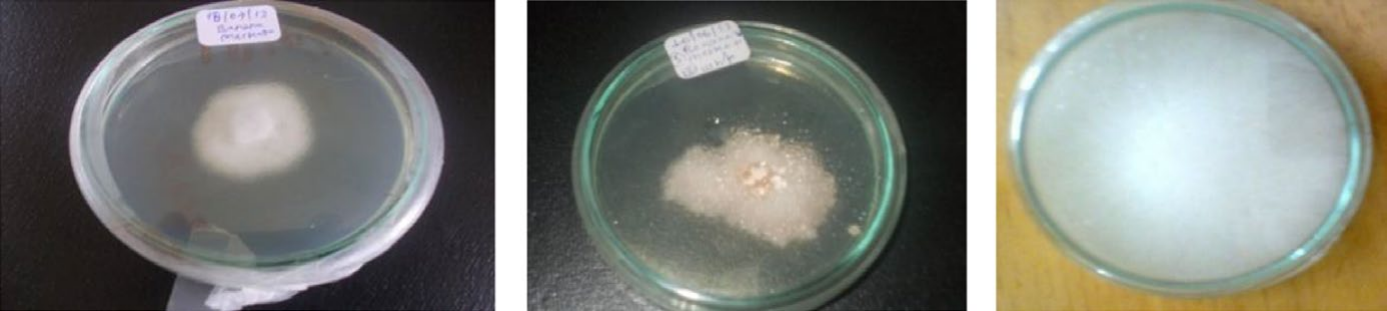
Pure cultures (After isolated from mother culture and incubated on PDA media)

**Figure 8:**
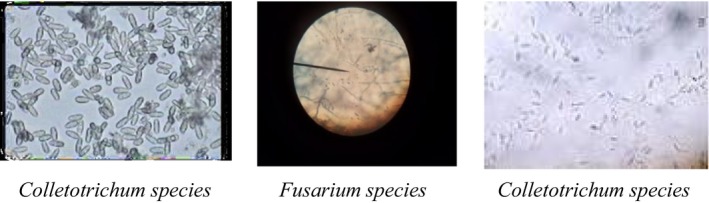
Morphological characteristics of fungal pathogens taken from pure culture

Growths of light pink colony of *Fusarium* were observed on sample taken from different parts of ripe fruits. This could be associated with high temperature used during ripening which may create more favorable condition for crown rot development, and *Fusarium* have been occasionally associated with crown rot disease of banana (Marin, Sutton, Blankenship, & Swallow, 1996). Sample taken from the study area is depicted in Figure 6, while Figures 7 and 8 show the pure cultures of fungal isolates and morphological characteristics of fungal pathogens isolated from banana fruit sample, respectively.

## 4 CONCLUSION

Banana fruits are highly susceptible to mechanical injury owing to their tender texture and high moisture content. If they are exposed to undesirable environmental conditions during handling, the tissue will soften and easily bruise, causing rapid microbial deterioration. In the study area, quality and safety assurance problems such as lack of temperature management, uniformity of quality within containers, sanitation problems in the market, transportation‐related problems, careless handling during loading and unloading were identified as the main factors, which favored fungal pathogen development and associated banana fruit losses. Disease intensity had similar trends with percentage of fruit damage with more damage, and infections were recorded in retailers’ shop. The percentages of fruit damage were as high as 56.2%, and the associated disease incidence and severity were 54.2% and 34%, respectively, in retailers’ shop. *Colletotrichum* spp. and *Fusarium spp* were identified as the most important fungal pathogens causing fruit loss in Jimma town of Bishishe market. Mechanical injury due to mishandling along supply chains and sanitary problem in the market could be the possible causes for observed fungal pathogens. In order to reduce mechanical injury and associated microbial deterioration, a close integration of all stakeholders along the value chain of banana fruits becomes necessary.

## References

[fsn3591-bib-0001] Babalola, J. B. (2011). World Bank Support for Nigerian Higher Education: Pleasure, Pains and Pathway towards a Knowledge Economy. *An Inaugural lecture delivered at the University of Ibadan*.

[fsn3591-bib-0002] Bailey, J. A. , & Jeger, M. J. (1992). Colletotrichum; biology, pathology and control (No. 04; QK625, B35.).

[fsn3591-bib-0003] Daniel, S. (1999). Banana in the southern region of Ethiopia (SRE) In Picq C., Fouré E. & Frison E. A. (Eds.), Bananas and food security 10‐14 November 1998 (pp. 119–128). Montpellier, France: INIBAP.

[fsn3591-bib-0004] Debela, A. , Daba, G. , Bane, D. , & Tolessa, K. (2011). Identification of major causes of postharvest losses among selected fruits in Jimma zone for proffering veritable solutions. International Journal of Current Research, 3(11), 040–043.

[fsn3591-bib-0005] Deka, B. C. , Choudhury, S. , Bhattacharyya, A. , Begum, K. H. , & Neog, M. (2006). Postharvest treatments for shelf life extension of banana under different storage environments. In *IV International Conference on Managing Quality in Chains‐The Integrated View on Fruits and Vegetables Quality* 712 (pp. 841–850).

[fsn3591-bib-0006] Duamkhanmanee, R. (2008). Natural essential oils from lemon grass (*Cymbopogon citratus*) to control postharvest anthracnose of mango fruit. International Journal of Biotechnology, 10(1), 104–108. https://doi.org/10.1504/IJBT.2008.017971

[fsn3591-bib-0007] Eduardo, K. (2012). Banana and plantain. San Jose, Costa Rica: Dole Fresh Fruit International Ltd http://www.ba.ars.usda.gov/hb66/banana.pdf

[fsn3591-bib-0008] Ellyn, S. (2011). What are the health benefits of banana bread? Retrieved from http://www.livestrong.com/article/268303-what-are-the-health-benefits-of-banana-bread/#ixzz2BVxmutdY

[fsn3591-bib-0009] El‐Naby, S. K. M. A. (2010). Effect of postharvest treatments on quality aspect of Maghrabi banana fruit. American‐Eurasian Journal of Agricultural and Environmental Science, 8(5), 582–587.

[fsn3591-bib-0010] FAO (2012). Food and Agriculture Organization of United Nations. Agriculture data base Prod STAT. Retrieved from http://faostat.fao.org/site/339/default.aspx

[fsn3591-bib-0011] FAOSTAT (2014). Banana Market Review and Statistics. Inter governmental Group on Bananas and Tropical Fruits. Market and Policy Analyses of Raw Materials, Horticulture and Tropical (RAMHOT) Products Team, Rome. Retrieved from http://faostat3.fao.org/home/index.html#DOWNLOAD

[fsn3591-bib-0012] Gultie, A. , Sahile, S. , & Subramanian, C. (2013). Assessment of fruit management in Gondar town markets of North Western Ethiopia. GJBAHS, 2(4), 4–8.

[fsn3591-bib-0013] Hailu, M. , Workneh, T. S. , & Belew, D. (2012). Effect of packaging materials on the quality of banana cultivars. African Journal of Agricultural Research, 7(7), 1226–1237.

[fsn3591-bib-0014] Idris, F. M. , Ibrahim, A. M. , & Forsido, S. F. (2015). Essential oils to control *Colletotrichum musae* in vitro and in vivo on banana fruits. American‐Eurasian Journal of Agricultural and Environmental Science, 15(3), 291–302.

[fsn3591-bib-0015] James, J. (2006). Overview of microbial hazards in fresh fruit and vegetables operations. Microbial Hazard Identification in Fresh Fruit and Vegetables, 1–36.

[fsn3591-bib-0016] Marasas, W. , Miller, D. , Riley, R. , & Visconti, A. (2001). Fumonisins‐occurrence, toxicology, metabolism and risk assessment In Fusarium. Summerell B. A., Leslie J. F., Backhouse D., Bryden W. & Burgess L. (Eds.), Paul E. Nelson Memorial Symposium (pp. 122–137). St. Paul, MN: APS Press.

[fsn3591-bib-0017] Marin, D. H. , Sutton, T. B. , Blankenship, M. , & Swallow, W. H. (1996). Pathogenicity of fungi associated with crown rot of bananas in Latin America on Grande Naine and disease‐resistant hybrid bananas. Plant Disease, 80(5), 525–528. https://doi.org/10.1094/PD-80-0525

[fsn3591-bib-0018] Mohapatra, D. , Mishra, S. , & Sutar, N. (2010). Banana and its by‐product utilisation: an overview.

[fsn3591-bib-0019] Mulualem, A. M. , Jema, H. , Kebede, W. , & Amare, A. (2015). Determinants of postharvest banana loss in the marketing chain of central Ethiopia. Food Science and Quality Management, 37, 52–63.

[fsn3591-bib-0020] Ogbo, E. M. , & Oyibo, A. E. (2008). Effects of three plants extract (*Ocimum gratissimum*,* Acalypha wilkesiana* and *Acalypha macrostachya*) on post harvest pathogen of *Persea americana* . Journal of Medicinal Plants Research, 2(11), 311–314.

[fsn3591-bib-0021] Olayemi, F. F. , Adegbola, J. A. , Bamishaiye, E. I. , & Awagu, E. F. (2012). Assessment of postharvest losses of some selected crops in eight local government areas of rivers state, Nigeria. Asian Journal of Rural Development, 2(1), 13–23.

[fsn3591-bib-0502] Robinson, J. C. , & Saúco, V. G. (2010). Bananas and plantains, Vol. 19 Cabi.

[fsn3591-bib-0022] Seifu, G.‐M. (1999). Banana: Production and utilization in Ethiopia (pp. 58). Research Report No. 35. Addis Ababa, Ethiopia: Ethiopian Institute of Agricultural Research Organization (EIAR).

[fsn3591-bib-0023] Singh, D. , & Sharma, R. R. (2007). Postharvest diseases of fruit and vegetables and their management. New Delhi, India: Daya Publishing House.

[fsn3591-bib-0024] Tilahun, W. S. , Osthoff, G. , & Steyn, M. S. (2012). Effects of preharvest treatment, disinfections and storage environment on quality of tomato. Journal of Food Science and Technology, 49(6), 685–694. https://doi.org/10.1007/s13197-011-0391-3 24293687 10.1007/s13197-011-0391-3 PMC3550837

[fsn3591-bib-0025] Vazquezshy, J. A. , Karina, D. , Adriano‐Anaya, M. D. L. , Salvador‐Figueroa, M. , & Ov, I. (2012). Sensory and physico‐chemical quality of banana fruits Grand Naine grown with biofertilizer. African Journal of Agricultural Research, 7(33), 4620–4626.

[fsn3591-bib-0026] Warton, M. A. , Wills, R. B. H. , & Ku, V. V. V. (2000). Ethylene levels associated with fruit and vegetables during marketing. Australian Journal of Experimental Agriculture, 40(3), 465–470. https://doi.org/10.1071/EA99125

[fsn3591-bib-0027] Woldu, Z. , Mohammed, A. , Belew, D. , Shumeta, Z. , & Bekele, A. (2015). Assessment of banana postharvest handling practices and losses in Ethiopia. Assessment, 5(17).

[fsn3591-bib-0503] Yamane, T. (1967). Statistics: An introductory analysis (No. HA29 Y2 1967).

[fsn3591-bib-0028] Zakaria, L. , Sahak, S. , Zakaria, M. , & Salleh, B. (2009). Characterisation of *Colletotrichum* associated with anthracnose of banana. Tropical Life Sciences Research, 20(2), 119. 24575184 PMC3819051

